# Where is crystallography going?

**DOI:** 10.1107/S2059798317016709

**Published:** 2018-02-01

**Authors:** Jonathan M. Grimes, David R. Hall, Alun W. Ashton, Gwyndaf Evans, Robin L. Owen, Armin Wagner, Katherine E. McAuley, Frank von Delft, Allen M. Orville, Thomas Sorensen, Martin A. Walsh, Helen M. Ginn, David I. Stuart

**Affiliations:** a Science Division, Diamond Light Source, Harwell Science and Innovation Campus, Didcot OX11 0DE, England; b Division of Structural Biology, Wellcome Centre for Human Genetics, Roosevelt Drive, Oxford OX3 7BN, England; c Research Complex at Harwell, Harwell Science and Innovation Campus, Didcot OX11 0FA, England; d Structural Genomics Consortium, Old Road Campus Research Building, Roosevelt Drive, Oxford OX3 7DQ, England

**Keywords:** macromolecular crystallography, synchrotrons, XFELs, electron diffraction, electron microscopy

## Abstract

Macromolecular crystallography (MX) has been a motor for biology for over half a century and this continues apace. A series of revolutions, including the production of recombinant proteins and cryo-crystallography, have meant that MX has repeatedly reinvented itself to dramatically increase its reach. Over the last 30 years synchrotron radiation has nucleated a succession of advances, ranging from detectors to optics and automation. These advances, in turn, open up opportunities. For instance, a further order of magnitude could perhaps be gained in signal to noise for general synchrotron experiments. In addition, X-ray free-electron lasers offer to capture fragments of reciprocal space without radiation damage, and open up the subpicosecond regime of protein dynamics and activity. But electrons have recently stolen the limelight: so is X-ray crystallography in rude health, or will imaging methods, especially single-particle electron microscopy, render it obsolete for the most interesting biology, whilst electron diffraction enables structure determination from even the smallest crystals? We will lay out some information to help you decide.

## Abstract 2

Macromolecular crystallography has provided results that underpin much biological discovery and there is still scope for further development; however, a revolution in electron imaging now means that it can also routinely provide detailed atomic-level descriptions. This article attempts to tease out where crystallography is going and consider what its place might be in the new landscape.

## 1. Introduction   

When one of us (DIS) decided to embark on protein crystallography some 40 years ago, he was warned by an aghast Head of Department and biophysicist (and Nobel prize winner) that this was a spent subject, the first protein structures having by then been established. Over the years other crises of confidence have occurred, but methods and technology have rescued the subject from the demise predicted by some of the greatest brains. For soluble proteins that crystallize readily, macromolecular crystallography (MX) is undoubtedly the method of choice for structure determination, with many preliminary structures being automatically generated within minutes of data collection at a modern synchrotron beamline. Now, however, the very technical advances that have propelled crystallography have also lifted other methods to the point where they can provide atomic level detail rather than molecular blobs. This includes imaging methods that do not rely on signal amplification through lattice formation. This ‘resolution revolution’ has transformed single-particle cryo-electron microscopy (cryo-EM) in the last five years (Kühlbrandt, 2014*a* ▸,*b*). X-ray crystallography is already well embedded in the drug-discovery process in the pharmaceutical industry and biotechnology companies. The expectation that the cryo-EM resolution revolution translates into an impact for these companies may have fuelled the acquisition of a leading EM equipment manufacturer, FEI, by the giant life-science company Thermo Fisher for $4.2bn in 2016, despite the lion’s share of FEI’s business probably being in the physical sciences at that point. This EM revolution came shortly after the demonstration of the potential of X-ray free-electron lasers (XFELs) to extend the scope of X-ray crystallography (Chapman *et al.*, 2011 ▸; Boutet *et al.*, 2012 ▸; Ginn *et al.*, 2015 ▸). New XFELS are now opening their doors (Ganter, 2010 ▸; Han *et al.*, 2012 ▸; Marx, 2017 ▸; Altarelli & Mancuso, 2014 ▸) and 26 more are planned (Zhao & Wang, 2010 ▸; Abbamonte *et al.*, 2015 ▸). Alongside these disruptive advances, new synchrotrons and upgrades of existing synchrotrons are planned. MX is dominant within the fraction of activity at a modern synchrotron devoted to life science (∼50% at the UK national source Diamond). Therefore, it is reasonable to scratch below the surface and ask where the science is going; do we have the balance right; are we missing opportunities? How much does structural biology justify a global investment of perhaps ten billion Euros in this enabling infrastructure? How should we divide the investment between the competing claims of X-rays and electrons, synchrotrons and XFELs? Some aspects of the substantial economic impact of structural biology have been discussed elsewhere (Berman *et al.*, 2016 ▸; Sullivan *et al.*, 2017 ▸), and overall it is clear that synchrotron MX is one of the great success stories for the impact of large infrastructure on basic and applied science, showing how technical innovation can enable discovery in biology. Accordingly, since we can predict everything except the future, now is certainly not the time to lose our nerve. Rather, we should think hard how we can work together to be cost-effective and optimize continued innovation of, and open access to, the most advanced technology, to maintain MX central to the exciting emerging opportunities for an integrated understanding of biological structure. In this paper, we give an overview of the state of the art in MX, with an eye to future prospects, especially focused around facilities at Diamond, the UK synchrotron, but incorporating information from other facilities as appropriate.

## 2. Inventions and reinventions   

**Figure 1:**
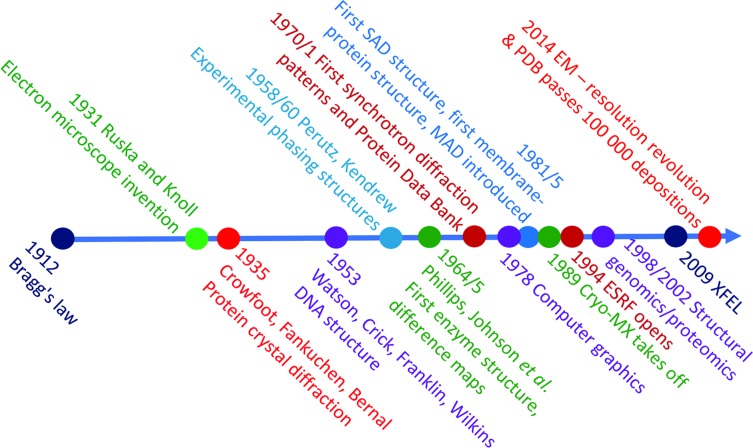
The development of macromolecular crystallography, with key highlights marked, over the last 100 years.

Fig. 1 ▸ indicates the timeline for some advances in MX. It is inevitably personal and many others have commented on these advances (see, for example, Su *et al.*, 2015 ▸; Hendrickson, 2013 ▸). What we would stress is that structural analysis can impact discovery in biology in more than one way. Mostly the impact has been *via* a reductionist, bottom-up approach: understand, dissect, analyse, reconstitute and analyse more. We will indicate below how this might be complemented by an *in vivo*, top-down approach, but there is still immense mileage in the reductionist approach applied to understanding isolated macromolecules and macromolecular complexes. For years synchrotron radiation has driven many aspects of the field, sometimes in obvious ways: for example, in international collaborations that have led to standards in sample handling such as the SPINE (Structural Proteomics in Europe, an EU project) pins for cryo-samples (Cipriani *et al.*, 2006 ▸). Less obvious perhaps is that the explosion in cryo-crystallography (Hope, 1988 ▸) was fuelled by the power of synchrotron beams that allowed the hundred-fold increase in crystal lifetime to be fully exploited. It is the combination of advances and opportunities that at some point tips interesting boutique methods into drivers for revolutionary change.

Diamond has now been operational for some ten years and has made an effort to collect data on the activity on the MX beamlines. These data, stored in the ISPyB database, provide interesting insights into a decade of developments. ISPyB was jointly developed between the ESRF and the UK before Diamond was operational; it was initially represented by the MRC-funded ESRF CRG beamline BM14 (Allan *et al.*, 2005 ▸; Delagenière *et al.*, 2011 ▸) and then further developed at Diamond (Fisher *et al.*, 2015 ▸). ISPyB itself is therefore an example of progress driven by international collaboration between scientific infrastructures, and provides an exemplar for data management which is now being extended to other types of beamlines at synchrotrons. The database is accessible through web services with interfaces *via* standard web browsers and mobile devices, tailored for beamline scientists and users, which allow the facile monitoring and reprocessing of data (Fisher *et al.*, 2015 ▸; Ginn *et al.*, 2014 ▸).

We present below data derived from the Diamond ISPyB database. One aspect that is not well tracked by ISPyB is the access mode. Remote access was introduced a number of years ago and is now likely to account for more than half of the MX activity at Diamond. In addition speed increases have led to access, primarily granted to groups of principal investigators (so-called BAGS, first introduced by the ESRF), being provided in smaller chunks, since even a few hours can yield a great deal of data. This in turn allows more frequent access. Changes will continue as automation facilitates the introduction of crystal-queuing systems and fully automated data collections, ready to absorb fragments of time that become free in the schedule.

## 3. Observations on recent trends and their extrapolation   

### 3.1. Speed of data collection   

**Figure 2:**
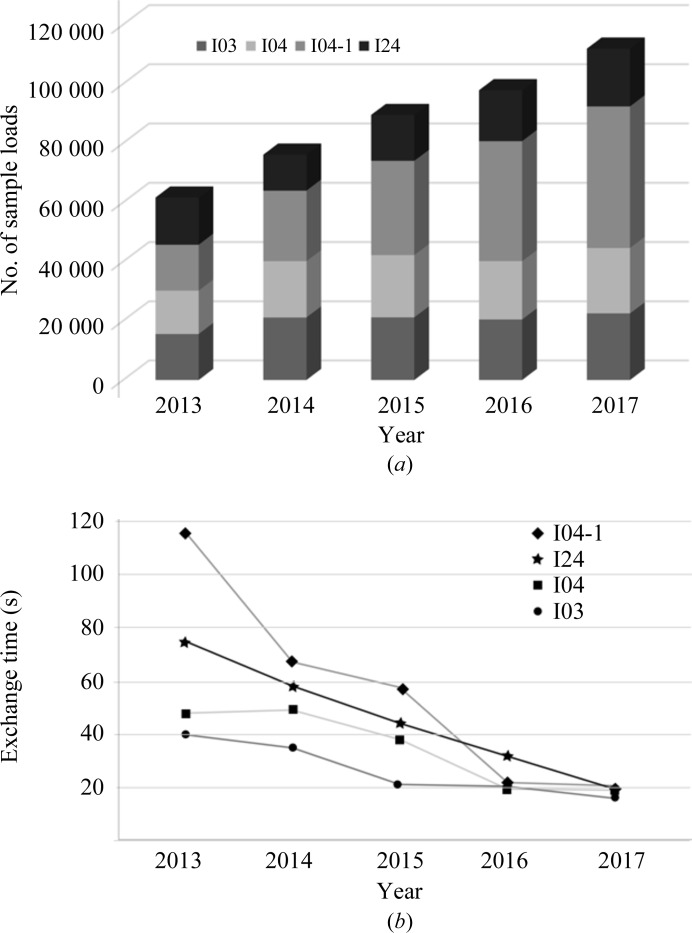
Automation statistics. (*a*) Total number of sample exchanges per year from 2013 to 2017 (the 2017 projection is based on current numbers). (*b*) Sample-exchange times per beamline per year.

**Figure 3:**
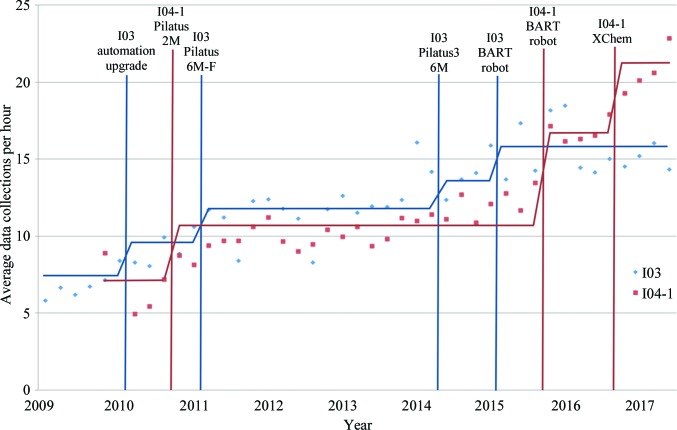
The evolution of data-collection rates at Diamond Light Source at two representative beamlines (I03 and I04-1). The chart shows the average data collections per hour *versus* the runs per year from 2010 to date. Significant upgrades to I03 in terms of automation and detectors are shown, and for I04-1 the installation of a detector and the BART robot and the opening of the XChem facility at Diamond.

The speed of data collection has increased in a stepwise fashion over the years, from ∼4 crystals per hour in 2007 to >20 crystals per hour in 2017 (Fig. 2 ▸). The reasons for this include (i) faster detectors, notably the Dectris pixel-array detectors (Broennimann *et al.*, 2006 ▸; Mueller *et al.*, 2012 ▸); (ii) automation, with sample exchange now taking little more than 10 s at Diamond, being far more reliable and the large storage dewars in the experimental hutches now allowing almost 600 crystals to be analysed without the need to enter the hutch (Fig. 2 ▸); and (iii) a range of additional improvements including simpler workflows, brighter beams and remote control of beamlines. Fig. 3 ▸ shows the data, with the impact of specific advances marked. We can expect further developments, including full automation with improved strategies, fast X-ray crystal centring, faster detectors and faster robotics (Bowler *et al.*, 2015 ▸). In practice, speed increases in individual components of the pipeline are not fully realised in terms of increased throughput, as new bottlenecks appear as others are eased. However, the improved efficiency makes it easier to screen crystals for more difficult problems and helps to routinely incorporate what would have been pathologies such as assembling data sets from multiple crystals. These should extend the reach of crystallography for the general user.

### 3.2. Reliability   

Reliability is distinct from speed, but is absolutely required if automation is to make a routine difference and be accepted by the user community. It has taken years to achieve the level of reliability that we now have at MX beamlines at Diamond. For example, the introduction of the Diamond BART sample changer reduced the critical failure rate of recoverable robot errors from 1.5 to 0.1%. This increased reliability now underpins all of the other advances.

### 3.3. Automated analysis   

**Figure 4:**
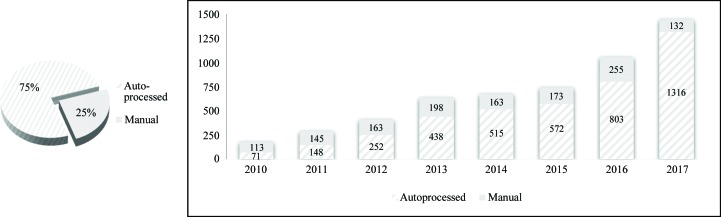
Matches between recorded autoprocessing results in Diamond’s ISPyB database and results accredited to Diamond beamlines in PDB depositions.

For eight years Diamond has implemented an automated pipeline for data analysis, derived from the work of Graeme Winter and Alun Ashton, in particular on *xia*2 (Winter & McAuley, 2011 ▸; Winter, Lobley *et al.*, 2013 ▸). This pipeline provides an initial near-real-time data analysis, followed by a more exhaustive automated analysis which provides the user with a best shot at data processing by the facility. A first-cut analysis is easy to justify, since it is helpful in planning experiments, and near-real-time feedback helps users to decide when to progress to the next stage. However, is the exhaustive analysis useful, or just a waste of cluster cores? User feedback suggests that this processing is roughly as good as many users achieve manually, and we suspect that it is frequently taken to be definitive. This notion is supported by the observation (Fig. 4 ▸) that 75% of deposited data from Diamond have unit-cell dimensions that agree on average to better than 0.2 Å with those from the autoprocessing.

What does the future hold? We can only guess, but we expect that automated procedures such as *DIALS* (Winter, Parkhurst *et al.*, 2013 ▸; Waterman *et al.*, 2016 ▸) will be able to (i) successfully process some currently intractable data sets; (ii) automatically apply improved strategies for data collection (Dauter, 2010 ▸; Waltersperger *et al.*, 2015 ▸; Finke *et al.*, 2016 ▸; Olieric *et al.*, 2016 ▸); (iii) automatically assemble and correctly dose-weight diffraction data sets from one to many overexposed data sets; (iv) provide improved estimates of difference signals (including anomalous scattering) through improved scaling methods; and (v) automatically generate optimal electron-density maps for ligands of interest (Pearce *et al.*, 2017 ▸).

### 3.4. Ease of structure solution (phase problem)   

As the database of solved structures grows (passing 100 000 depositions in 2014), we would expect that an increasing fraction of unknown structures will be solvable by molecular replacement. However, the data from the last ten years at Diamond suggest that the ambition of the community has grown to offset this, and about 10% of activity remains focused on phase determination. Most of this is single-wavelength anomalous dispersion (SAD) phasing, with some multiple-wavelength anomalous dispersion (MAD) and classic isomorphous replacement. Clearly, for certain systems the phase problem is still a significant hurdle and Diamond is attempting to alleviate this by extending the power of long-wavelength MX through the construction of beamline I23, as discussed below.

### 3.5. Molecular size and resolution   

Interestingly, the data show that there has been a slight increase in the average size of the molecule(s) under analysis, from ∼90 kDa in 2008 to ∼100 kDa in 2016. This reflects the increasing reach of MX. Alongside this, the average resolution of the analyses has improved marginally (2.29 Å in 2008, 2.17 Å in 2016). If the crystal size, solvent content, *B* factor *etc.* remain largely the same, the average scattered intensity will scale with the inverse of molecular weight, so it is a significant achievement to maintain data quality in the face of increasing molecular weight.

### 3.6. Crystal and beam size   

When Diamond started in 2007, the three so-called Phase I MX beamlines, I02, I03 and I04 (http://www.diamond.ac.uk/Beamlines/Mx.html), provided a beam ∼80 µm across, probably somewhat smaller than the average crystal size at that time. Soon after, I24 came online as the first tuneable microfocus beamline, routinely providing a 10 µm diameter beam (Evans *et al.*, 2007 ▸; Evans, Axford & Owen, 2011 ▸). Since then, this beamline has been in heavy demand, especially, it seems, for membrane-protein work. The average crystal size across all of the MX beamlines has certainly decreased, although this is difficult to quantify, and now beam sizes can be routinely tailored to match crystal size. The use of microcrystals is a major synchrotron success story, and we discuss the future of this below.

### 3.7. Crystal presentation   

For the last ten years, the vast majority of MX data sets collected have maintained the crystals at ∼100 K during exposure, usually bathed in a stream of cold gaseous nitrogen. The crystals are manually mounted in loops, meshes, grids *etc.*, which are now made commercially, and attached to SPINE standard pins (Cipriani *et al.*, 2006 ▸). This is compatible with current automated methods for sample presentation, but has a number of drawbacks. It usually relies on mounting single crystals within individual loops, a time-consuming process which remains largely manual and requires some expertise by the user. The loops have often been much larger than the crystals, making simple machine-vision algorithms inadequate to properly centre the crystals within the beam and degrading the signal to noise of the diffraction data. Commercial loops are now available in a range of sizes, and matching the loop to the crystal size can allow rapid automated data collection and optimized signal to noise (Sanishvili *et al.*, 2008 ▸). This has proven to be particularly effective for the fragment-screening service XChem (http://www.diamond.ac.uk/Beamlines/Mx/Fragment-Screening.html). If you are a user and do not loop-match, we would suggest that you try it!

To automate crystal mounting, some additional challenges must be overcome. The crystal usually needs cryoprotection (soaking in a liquor spiked with cryoprotectants such as glycerol, polyethylene glycol *etc.*), which requires time and experimentation to determine the correct cocktail. The question of whether this soaking and cooling damages the crystal lattice can be conveniently addressed by illuminating the crystal *in situ* in the crystallization drop at room temperature. Such room-temperature experiments provide a gold-standard evaluation of the inherent quality of the crystal lattice. Cryoprotection is easier for small crystals, which probably do not require the addition of cryoprotectants if all of the surrounding liquor can be quickly removed immediately before rapidly cooling. This method is used in the innovative CrystalDirect robot developed at EMBL Grenoble, with a high success rate reported (Cipriani *et al.*, 2012 ▸). In addition CrystalDirect completely automates crystal mounting, eliminating the substantial overhead of mounting single crystals manually into individual loops, and achieves a mounting rate that roughly matches the consumption rate of a modern beamline. A much cheaper semi-automatic system is the Shifter, which was developed at the Structual Genomics Consortium (SGC) and Diamond and has now been commercialized (https://oxfordlabtech.wordpress.com/shifter/). With practice, a user can comfortably mount some 100 crystals per hour with the Shifter, which is available as part of the XChem offering at Diamond (http://www.diamond.ac.uk/Beamlines/Mx/Fragment-Screening.html).

Such technologies will shift the bottleneck in data collection, likely to the time taken for sample exchange and centring. However, there is no reason why several crystals should not be mounted in a single loop, mesh or grid; indeed, for microcrystals a large number can be presented on a single mesh, allowing potentially highly efficient data collection (Axford *et al.*, 2014 ▸). Alternative methods are being investigated, for instance in which several crystals can be loaded in a row on a purpose-fabricated mount, or data collection from crystals *in situ* in the crystallization drop, which eliminates the need to mount crystals. Several beamlines offer *in situ* data collection, with early pioneering implementations at the ESRF and SLS (Bingel-Erlenmeyer *et al.*, 2011 ▸; le Maire *et al.*, 2011 ▸). The I24 and I03 beamlines at Diamond now offer high-precision stages which allow the collection of good-quality data from even small crystals using specially designed SBS-format crystallization plates which reduce X-ray scatter from the plate and cover material. Structure determination as well as crystal screening is then quite feasible (Owen *et al.*, 2014 ▸; Axford *et al.*, 2012 ▸). To build on this, an ultrabright modular automated beamline optimized for room-temperature data collection (VMXi) has been developed at Diamond and its potential impact is discussed below.

A particular niche has been the collection of room-temperature data from virus crystals, which has provided several high-resolution structures (Wang *et al.*, 2012 ▸, 2015 ▸; Ren *et al.*, 2013 ▸). However, the advent of the latest methods in electron microscopy has largely eliminated this niche (the likely effects of EM developments on the future of MX are further discussed below). A more radical alternative method of sample presentation, so-called ‘serial crystallography’, is discussed below.

## 4. Significant upcoming opportunities   

The following sections are not exhaustive, and highlight developments at Diamond.

### 4.1. VMXi   

**Figure 5:**
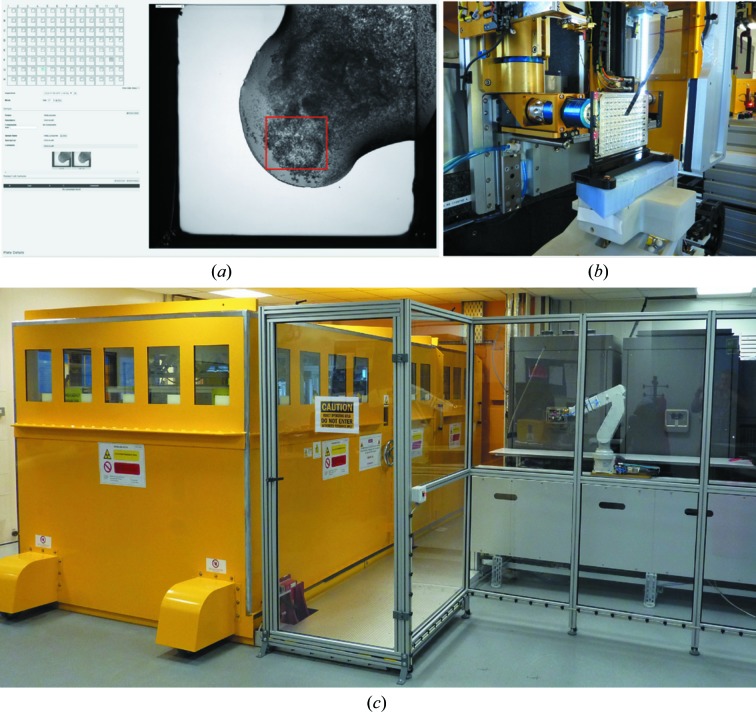
VMXi user interface in SynchWeb. The marked area (red line) is an example of an area selected for X-ray scanning on the beamline. (*b*) View of the VMXi sample position, with the plate mounted in front of the beamline imaging system. (*c*) VMXi mini-hutch, crystallization-plate storage and external robotic arm for plate transfer from storage to beamline.

VMXi will offer fully automatic operation and deploys a double-multilayer monochromator, increasing the energy bandpass (essentially capturing the full width of the undulator peak), to provide some 10^14^ photons s^−1^ at 13 keV (∼0.95 Å wavelength). The beamline is designed for room-temperature measurements, where the generally accepted 300 kGy dose limit will be reached within ∼30–100 µs using the unatten­uated, focused beam. The use of a high-speed Dectris EIGER detector (routinely operating at up to 750 Hz) will allow the very rapid interrogation of small crystals at room temperature. Coupled with the automated light-microscope imaging of the (diffraction-optimized) crystallization plate and web-based markup, the ease of queuing crystals for in-plate X-ray analysis and robotic plate transfer (Fig. 5 ▸), it is hoped to cut the time for crystal optimization for challenging projects (especially for membrane proteins, in conjunction with the Membrane Protein Laboratory; http://www.diamond.ac.uk/Beamlines/Mx/MPL.html) by offering essentially on-demand X-ray analysis, rather than waiting for scheduled beamtime. VMXi may also, by complete X-ray scanning of drops before plate removal, answer a question that nags at many of us, which is: are there diffracting microcrystalline slurries in trays that are missed by conventional methods of searching for crystals? Furthermore, as a very bright beamline with a modular endstation design VMXi will be a great testbed for room-temperature crystallography of other flavours, in particular serial crystallography (see below) and time-resolved MX studies, opening up the analysis of systems operating in time regimes of microseconds/milliseconds and longer, complementing XFEL sources, where the LCLS and European XFELs deliver 10^12^–10^13^ photons per 50 fs pulse in a ≤5 × 5 µm size beam, with a 5 to 1 × 10^−3^ bandpass in self-amplified spontaneous emission (SASE) mode. Finally, routine access to room-temperature structures will allow systematic comparisons with cryogenic structures and experimental mapping of conformational landscapes (up to 35% of side chains have been reported to be remodelled at cryo-temperatures; Fraser *et al.*, 2011 ▸).

### 4.2. I23   

**Figure 6:**
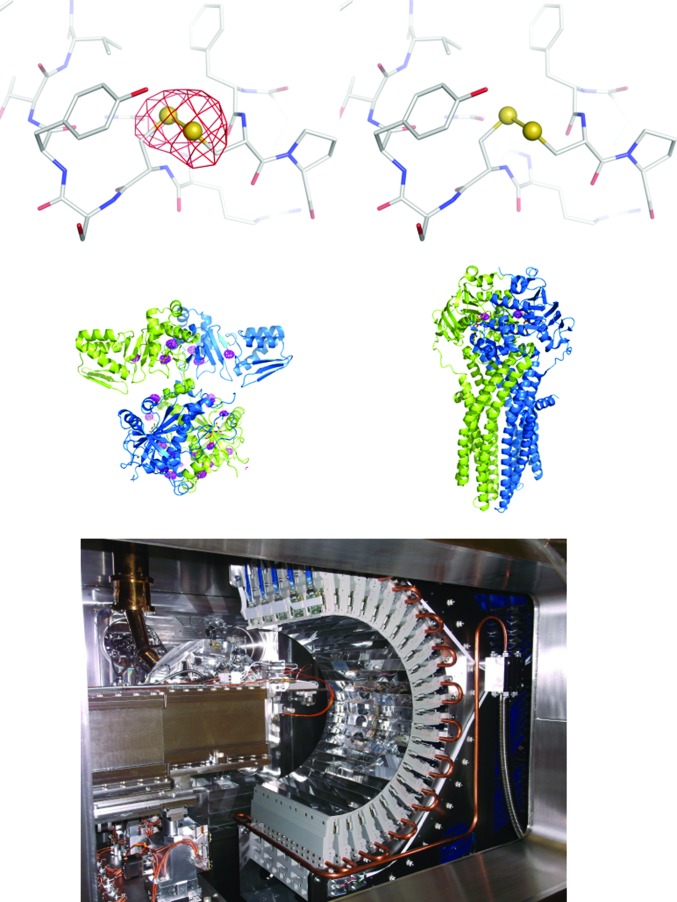
Recent I23 results and detector. Top panel, anomalous difference Fourier maps at 4σ for thaumatin at λ = 4.96 Å (*E* = 2.5 keV, left) and λ = 5.17 Å (*E* = 2.4 keV, right). Peaks disappear beyond the edge. Middle left, anomalous difference Fourier map at 4σ for cyanobactin oxidase which was solved by S-SAD (Bent *et al.*, 2016 ▸) with λ = 3.1 Å; middle right, anomalous difference Fourier map at 1σ for ADP-vanadate in the ABC transporter collected at λ = 2.26 Å (Bountra *et al.*, 2017 ▸). The bottom panel shows the 12 Mpixel PILATUS detector inside the (opened) vacuum endstation.

I23 is the first, and so far the only, synchrotron beamline designed to capture data from a soft X-ray beam by operating in vacuum (Wagner *et al.*, 2016 ▸; Aurelius *et al.*, 2016 ▸). It has been a tremendous challenge to construct and, although now operational, it will remain in an optimization phase for some time. The beamline has already solved some tough phasing problems and can be tuned to below the 2.472 keV (5.02 Å wavelength) *K* edge of sulfur (the limit of the beamline is about 2.1 keV; 5.9 Å wavelength), making it ideal for measuring anomalous scattering signals from lighter atoms (Fig. 6 ▸). What have we learnt? It should not have been a surprise to discover that conventional beamlines introduce significant background scatter, which tends to drown out weak anomalous scattering signals. Typically I23 can deliver a tenfold improvement in signal to noise for weaker reflections. As a result, phasing from endogenous atoms (for example sulfur) becomes far more tractable, since the soft X-rays stimulate an enhanced anomalous signal, which can then be measured more accurately owing to the improvement in signal to noise. For most protein crystals, the phase problem should therefore be eliminated. Other interesting experiments also open up on I23; for instance, by probing the anomalous signal of bound ions it is possible to distinguish potassium from sodium or locate S and P atoms to assist with model building at low resolution.

### 4.3. Microfocus   

Microfocus MX originated at EMBL Grenoble/ESRF with the implementation of the microdiffractometer (Perrakis *et al.*, 1999 ▸), and many synchrotrons now have dedicated microfocus beamlines. The first tuneable microfocus beamline was I24 at Diamond, designed by Gwyndaf Evans (Evans *et al.*, 2007 ▸; Evans, Axford, Waterman *et al.*, 2011 ▸), and for some time this has been the most oversubscribed of the Diamond MX beamlines. To help alleviate the demand for microfocus beamtime, beamline I04 installed the Diamond-designed F-Switch (Duller *et al.*, 2016 ▸), a device that uses variable arrays of two-dimensional refractive lenses in place of conventional focusing mirrors. This enables users to quickly dial up a focused beam in steps ranging from 5 to 100 µm over the energy range 6–18 keV, so that microfocus beam experiments can be dispersed amongst more routine data collections.

The smallest crystals that have been successfully solved *de novo* on I24 are ∼3 µm in all dimensions (Ji *et al.*, 2015 ▸). For such tiny crystals the endstation must be designed to optimize the region around the crystal position (remember that 1 mm of air absorbs X-rays similarly to 1 µm of protein crystal). VMXm (also designed by Gwyndaf Evans) is likely to be the first beamline expressly targeting submicrometre crystals and beams. This beamline builds on experience from I24 and I23 and will incorporate a modestly sized evacuated sample environment housing a scanning electron microscope for crystal visualization and very high precision goniometer. The X-ray optics present severe challenges, especially in terms of stability, and VMXm incorporates extensive use of interferometry to monitor beam and X-ray mirror positions and uses feedback loops to maintain stability. Another innovation is the use of a focusing mirror divided into a number of laterally separated lanes, each of which has a profile calculated to shape the X-ray beam to a particular size at the focal point, allowing rapid changes in beamsize (∼1 s; Laundy *et al.*, 2016 ▸).

### 4.4. XChem   

XChem, implemented as a service on beamline I04-1 and developed in partnership with the SGC, Oxford, is designed to maximize the potential of a fixed-wavelength beamline by integrating it into a highly optimized pipeline for crystallo­graphic fragment screening. In practice, this has involved developing sets of fragments (rather less than 1000 in number) which are well characterized and chosen to be ‘poised’ for chemical elaboration (Cox *et al.*, 2016 ▸). These compounds are validated and formulated for crystal soaking, which is achieved using an acoustic drop ejector (Echo 550; Labcyte, 170 Rose Orchard Way, San Jose, CA 95134, USA) to propel drops of compound in DMSO into the crystallization drops (Collins *et al.*, 2017 ▸). This can be accomplished in less than 1 min for a 96-well plate. After some time soaking, crystals are mounted semi-automatically using the Shifter (see above) and stored in unipucks (http://smb.slac.stanford.edu/robosync/Universal_Puck) ready for data collection. Loops are matched to crystal size so that automated centring using a Keyence machine-vision system, followed by fast image analysis of the video feed of the on-axis crystal viewing microscope, is very successful. Combining this with the in-house crystal-changing robotics described above allows up to 1000 crystals (and data sets) to be analysed in 2 d. Acquiring and automatically processing such a mass of data sets opens the way for innovative analyses, and an elegant example of this is the *PanDDA* software (Pearce *et al.*, 2017 ▸), which takes all data sets into account to map out in real space where significant changes occur and allows very sensitive detection of binding sites.

### 4.5. Serial crystallography   

**Figure 7:**
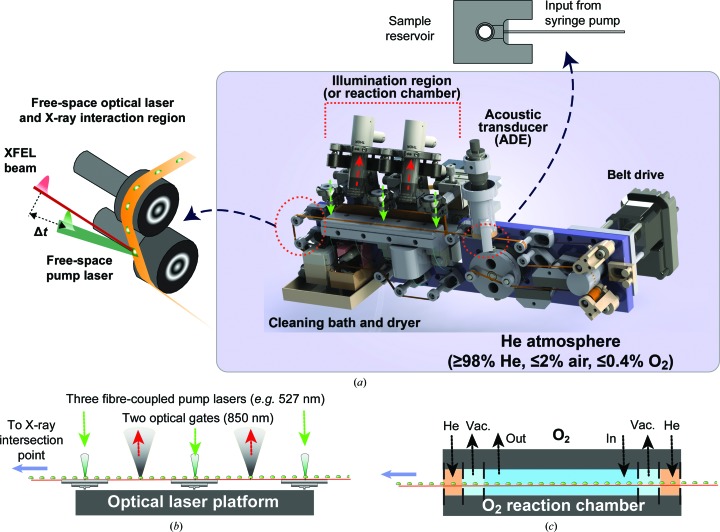
The acoustic injector conveyor-belt system supports several types of time-resolved serial femtosecond crystallography experiments. (*a*) The schematic plan designed for the MFX hutch at the LCLS. (*b*) The optical laser platform enables pump–probe time-resolved SFX with three illumination fibres and two optical gates. A laser intersects with the sample and XFEL pulse in the interaction region. (*c*) A schematic plan for the O_2_ reaction chamber used in September 2016 during experiment LN83, where droplets on a tape pass through small orifices separating He, partial vacuum and 100% O_2_ chambers (Fuller *et al.*, 2017 ▸).

Serial crystallography takes data-collection rates to another level. Essentially, the term refers to methods that place many crystals in the beam in rapid succession without regard to their orientation and collect a short-exposure still diffraction image from each crystal. This is simply the American method, developed many years ago for virus crystallography, on steroids (Rossmann & Erickson, 1983 ▸). Such methods can be surprisingly efficient in collecting a relatively complete data set (Fry *et al.*, 1999 ▸). The method works perfectly with XFEL beams, which damage crystals in tens of femtoseconds, rendering conventional multi-image data collection unfeasible. Describing the full gamut of serial methods that are being developed is beyond the scope of this article, but in brief they can position the crystals in the beam as part of a high-speed liquid jet (the so-called gas dynamic virtual nozzle) moving at ∼10 m s^−1^ (Weierstall *et al.*, 2012 ▸; Oberthuer *et al.*, 2017 ▸), or within a viscous extrusion moving at perhaps a few millimetres per minute (Weierstall *et al.*, 2014 ▸; Botha *et al.*, 2015 ▸). Alternatively, droplets might be used, made either by a nozzle (Sierra *et al.*, 2012 ▸) or using an acoustic drop ejector (as used in the XChem workflow), to deposit the crystals on a tape drive which moves the crystals through interaction points (Roessler *et al.*, 2016 ▸; Fuller *et al.*, 2017 ▸; Fig. 7 ▸). Recently, there has been good progress in using silicon chips fabricated with small depressions which can harbour crystals. Each chip can contain many thousands of depressions spaced closely to­gether. Combining the chip with high-speed and high-precision piezo translation stages can allow collection at a rate of ∼100 Hz with a high crystal hit rate (Owen *et al.*, 2017 ▸). The use of crystalline silicon means that the background can be reduced, as long as the silicon Bragg reflections, which gather the vast majority of the scattering from the chip substrate, do not fall on the detector. Groups in Hamburg, Toronto and Diamond have been particularly active in developing these methods (Mueller *et al.*, 2015 ▸; Roedig *et al.*, 2015 ▸, 2017 ▸; Hunter *et al.*, 2014 ▸; Cohen *et al.*, 2014 ▸; Oghbaey *et al.*, 2016 ▸).

What the role of serial crystallography will be at synchrotron sources remains unclear, since it can be very demanding in terms of crystal numbers, but at a beamline such as VMXi, its use for time-resolved work and room-temperature data collection where crystal lifetime is very limited may prove to be of real value.

### 4.6. XFELs   

MX with XFEL radiation hit the headlines in 2011 with real protein structure information (Chapman *et al.*, 2011 ▸). The key advantages are brightness (perhaps eight orders of magnitude higher than that at synchrotrons) and time structure (pulses of usually tens of femtoseconds). The short timescale allows the problem of radiation damage to be largely side-stepped, which is a winner for small weakly diffracting crystals (however, see Nass *et al.*, 2015 ▸), and very fast dynamics can be tackled (Nango *et al.*, 2016 ▸; Barends *et al.*, 2015 ▸; Young *et al.*, 2016 ▸). Where XFELs have made significant impact to date is in the study of microcrystals (enabling structures where microfocus synchrotron beamlines had failed; Ginn *et al.*, 2015 ▸; Gati *et al.*, 2017 ▸; Stagno *et al.*, 2017 ▸); membrane proteins, especially GPCRs, where crystals are frequently small and weakly diffracting (Liu *et al.*, 2013 ▸; Kang *et al.*, 2015 ▸); and dynamic studies, for instance the structures of different light-activated states of PSII at room temperature (Young *et al.*, 2016 ▸; Fuller *et al.*, 2017 ▸; Suga *et al.*, 2017 ▸), myoglobin (Barends *et al.*, 2015 ▸) and photoactive yellow protein (Tenboer *et al.*, 2014 ▸; Pande *et al.*, 2016 ▸). The current XFELs [Linac Coherent Light Source (LCLS) and SPring-8 Angstrom Compact Free Electron Laser (SACLA)] are heavily oversubscribed and more than an order of magnitude more expensive per second of beamtime than a synchrotron. Using them effectively is therefore a challenge, and for now the studies have been limited to a few otherwise intractable high-value experiments. However, three new facilities are coming on line in 2017 (PAL XFEL in Korea, the European XFEL in Germany and the SwissFEL in Switzerland). Each of these will support one or more instruments for structural biology. Capacity can be further increased by refocusing the majority of X-rays that simply pass directly through the sample/interaction region for use in a second, downstream instrument. Thus, the SPB/SFX instrument at the European XFEL and the CXI instrument at the LCLS can run two experiments simultaneously. Finally, XFELs are moving from build-it-yourself experiments to more robust (synchrotron-like) user facilities. To help users make the most of expanding XFEL facilities, the XFEL Hub at Diamond was created in 2015 following a beamline model. The Hub conducts research and development in time-resolved serial MX, on-demand sample-delivery methods and facilitates technology transfer between XFEL, synchrotron and cryo-EM facilities. The Hub also helps structural biologists with all aspects of XFEL-based research, ranging from proposal preparations through to publications (http://www.diamond.ac.uk/Beamlines/Mx/XFEL-Hub.html).

## 5. The ultimate MX experiment   

**Figure 8:**
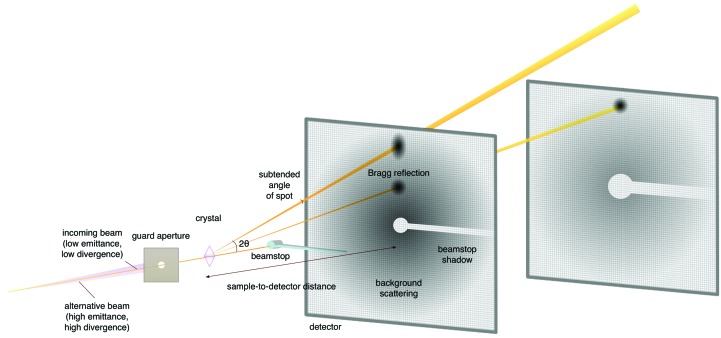
A classic MX experiment. Many of the components are described in the main text of the article. Note in particular that two possible experimental setups are superimposed: one with the detector close in and the second with it pushed back. Owing to the Bragg reflections expanding from an effective source position many metres upstream of the crystal, the spot shape and intensity are not very different in the two experiments, whereas the background scattering, which arises largely from scattering from disordered crystal components, material around the crystal and air scattering from the direct beam between the guard aperture and the backstop, falls off as the square of the distance from that point to the detector and is therefore dramatically reduced at longer crystal-to-detector distances.

The preceeding sections report on progress and some plans for the future, but the discussions are phenomenological and illustrate targeted developments. In this section we will approach MX from a different direction, that of optimizing a general experiment, and then show how the developments discussed above also fit with the underpinning aim of approaching the ideal MX experiment, and indicate how far we might approach this ideal. An MX experiment can be represented by classical scattering equations, and although these look rather intimidating the underlying physical principles are simple. For the ultimate experiment let us assume that we have a perfect detector, no background scatter and a perfect synchrotron beam. We also assume that the experiment will be radiation-damage limited, which may not always be the case in serial crystallography. The accuracy of a scattering observation is limited by inherent shot noise, which means that the error is the square root of the total photon count. Even the most pessimistic crystallographer would accept that intensities of two standard deviations are useful, and so the minimum number of counts needed per reflection to achieve this is just four. In practice, inspection of the raw data for a spot with a processed intensity of 2σ will show that the actual number of counts in the spot is orders of magnitude greater than this. The principle cause of this mismatch is the background scattering from extraneous items, such as the crystal mount and air in the path of the beam, which adds to the total count in each pixel, thereby increasing the error but not the signal. Consider a spot spread over ten pixels with a background level of 40 counts per pixel. The error arising from the background alone is (400)^1/2^, *i.e.* 20, so in order to achieve a final signal-to-noise ratio of 2 we would need in excess of 40 counts in the signal. It is even worse than this in practice since we cannot know exactly what the background level is, and we therefore introduce further error when we try to subtract the background by using an estimation. Therefore, a near-ideal experiment will concentrate the Bragg scattering into the narrowest ray possible, which falls easily within a single pixel of the detector. The pixel should subtend a small solid angle with the crystal, *i.e.* it should be small and the detector should be pushed as far back as possible without losing high-resolution data. The background in a fixed-size pixel reduces as the square of the distance from the crystal, whereas for almost parallel Bragg scattering the spot will effectively retain the same size and brightness as the detector is pushed back. In practice beam divergence, crystal mosaic spread and the energy spread of the beam will combine to somewhat disperse the Bragg spot (Fig. 8 ▸).

In real experiments compromises have to be made, but it is useful to map recent developments and potential improvements onto the ideal experiment and understand the reasons for the compromises and how close we might get to the ideal experiment. The driver is that although cryo-crystals have a lifetime in the beam of approximately 100 times that at room temperature, radiation damage still limits the data that can be obtained from a single crystal. In 2010, James Holton published an excellent paper bringing together many of the relevant bits of theory to enquire what the ideal MX experiment might achieve and found that current beamlines required 15–700 times the scattering power of the ideal experiment (Holton & Frankel, 2010 ▸). The scattering is directly proportional to the crystal volume and Holton estimated that (neglecting photoelectric escape enhancements; discussed below) for a spherical idealized lysozyme crystal a diameter of 1.8 µm was the smallest size that could yield a complete data set providing 2 Å resolution data of mean intensity 2σ. As we noted above, Bragg intensities are reduced in proportion to the molecular weight of the protein or complex, so that for a large macromolecular complex the crystal volume required might be several hundred times greater. Serious inroads have been made into the terrible discrepancy between the ideal and reality since the 2010 Holton paper, and it is worth reminding ourselves of the sources of the problem and the steps taken to mitigate it.

### 5.1. Beam size and divergence   

It is important to match the incident beam size to that of the crystal. Illuminating excess material will simply inflate the background scatter and add no signal to the Bragg peaks. On modern beamlines it is often easy to quickly dial up beam sizes to match them, on the screen, to crystal images. Microbeams of 5 × 5 µm can be defined on I24 using microapertures and these can be crucial in reducing the background signal, for instance in structures derived by in-cellular crystallography (Baskaran *et al.*, 2015 ▸). However, it is still rare to dynamically change the beam size as the crystal rotates. In principle, the solution is simple. The beamline software should establish a three-dimensional model of the crystal as a preliminary to the diffraction experiment and the beam size should be adjusted to match the projection of this model along the beam as the crystal rotates. A simple partial solution is to match the loop to the crystal size (as implemented in XChem).

A second beam parameter is the divergence. In principle the ideal beam would actually be slightly convergent, to both optimally illuminate the crystal and focus the beam at the detector within a single pixel. As long as the beam size at the detector is less than the pixel size there is little to be gained by making it smaller, and the detector should then be placed as far back as possible whilst collecting all of the useful data and maintaining this criterion. However, microfocus beams tend to be much smaller than current detector pixels and a greater level of divergence has often been taken as a reasonable compromise. This is one area where the continual improvement in mirror and monochromator technology, combined with the greatly reduced emittance (greater brightness) of the new generation of synchrotron lattices (magnet arrangements), can have a positive impact. The reduced emittance is the main driver for the new round of lattices being built or planned. For instance, Diamond, which has been in operation for ten years and was class-leading when it was built, has a horizontal emittance of ∼3 nm rad, which is more than ten times that of MAX IV at Lund, Sweden, which is just coming online. Reduced emittance, which translates into less divergent, smaller, brighter beams, is the major advantage for MX from these new machines (including the new lattice for the ESRF and APS, and the proposed upgrades of SLS and Diamond).

### 5.2. Beam energy   

We have seen above that to deliver a general phasing vehicle, a beamline producing lower energy, so-called ‘softer’ X-rays, is ideal. However, what is the ideal energy to maximize the amount of diffraction data that can be gathered from a single precious crystal? Here again, Holton has usefully collated the data (Holton & Frankel, 2010 ▸). For larger crystals, where the photoelectron-escape effect can be ignored, the ideal appears to be around 25–30 keV, which is much higher than currently used routinely for MX (corresponding to a wavelength of less than 0.5 Å). Until recently, it has not been possible to purchase detectors that operate competitively at this energy (for example in terms of number of pixels and detector quantum efficiency) compared with a PILATUS or EIGER (Dectris) operating at ∼12 keV. Furthermore, medium-energy machines such as Diamond have not been equipped with insertion devices that could provide a competitively bright beam at such energy. However, both of these constraints are being relaxed. A new generation of detectors using a sensor made out of more X-ray-absorbent material (*e.g.* CdTe; so-called high-*Z* detectors) is now on the market (*e.g.* the Dectris PILATUS3 X CdTe) and undulators with increased magnetic field strength, achieved by using either cryo-permanent magnet technology (Benabderrahmane *et al.*, 2011 ▸) or superconducting magnet technology (Ivanyushenkov *et al.*, 2013 ▸), are starting to become routinely available. Putting these two technologies together, we might expect a modest improvement in data yield before destruction and the virtual elimination of absorption effects. There is little data to substantiate this yet, but preliminary experiments suggest a gain of perhaps 30% (Meents & Wagner, unpublished work). Furthermore, the mean path length for photoelectron damage increases with increasing photon energy, and so this might give a substantial benefit for microcrystals (see below).

### 5.3. Photoelectron escape   

Although the mechanisms of radiation damage remain unclear (Garman & Nave, 2009 ▸), it is thought to be owing in large part to the photoelectrons that are released when a photon interacts with an atom. The energy of the photoelectron is gradually reduced as it moves through the crystal and the most damaging effects are thought to arise towards the end of its path. Naturally higher energy photons will have a longer average path length. The effect of this on crystal lifetime was first addressed by Nave & Hill (2005 ▸), and an alternative, simplified, model was proposed by Holton & Frankel (2010 ▸). The effect would mean that for, say, a 1 µm crystal and 12 keV X-rays most of the damage should occur outside the crystal, and might decrease the diameter of the crystal required for a complete data set by a factor of ∼3 (Holton & Frankel, 2010 ▸; Sanishvili *et al.*, 2008 ▸). It has proved frustratingly hard to fully verify this effect; however, a beamline such as VMXm is ideally placed to investigate and fully exploit it.

### 5.4. From guard aperture to backstop   

Ideally, the critical interaction region from guard aperture to backstop (Fig. 8 ▸) would be in a vacuum, and as noted above results from I23 have demonstrated that this allows a dramatic reduction in background scatter. We expect an increasing use of in-vacuum endstations where it is critical to optimize the signal to noise (*e.g.* VMXm). As for the crystal environment, we have already noted efforts to minimize the surrounding scattering material (for example loop-matching and liquid removal in CrystalDirect), whilst liquid jets and LCP extruders remain suboptimal and still need work to improve the extraneous scattering. In contrast, suitably prepared crystalline silicon chips offer a near-perfect mount, and in principle can act as weak collimators to help clean up the beam. One aspect worth exploring is the possibility of crystal ‘machining’. This could be achieved either with an ablative laser or ion beam (Rigort & Plitzko, 2015 ▸; Schaffer *et al.*, 2017 ▸), and could mitigate absorption differences between reflections when collecting data using longer wavelength X-rays. Note that away from absorption edges absorption increases roughly in proportion to the cube of the wavelength, and so might be ∼50-fold worse on I23 than on a conventional beamline, essentially forcing the use of microcrystals. Alternatively, sample machining might just be used to ablate extraneous scattering/absorbing material.

### 5.5. Goniometry   

Current goniometry is capable of maintaining a micrometre-sized crystal within a microbeam as it rotates and modern multi-axis goniometers introduce relatively little inflation of the sphere of confusion (for example, the Smargon device; http://www.smaract.com/products/6d-smargon-goniometer/), and so it becomes practical to automatically collect multiple data sweeps in different orientations to cancel out certain systematic errors. The increased frame rate of detectors and the speed and precision of goniometer rotations also means that finer slicing of the reflection around the rotation axis is possible, which should increase the accuracy of the measurement (at least until the rocking width of the reflection is heavily oversampled). The reflection rocking width will depend on (i) crystal parameters, notably mosaic spread, which might be reduced by regimes such as that employed by CrystalDirect (see above); (ii) crystal size (spots will be inflated for very small crystals; although this effect may be important for XFEL data, it is usually insignificant for synchrotron data collection); and (iii) beam parameters: divergence leading to an increase in the reflecting range. As always, optimum signal to noise will be achieved with the smallest possible spot. Note that the use of beams with a larger energy bandwidth may limit the signal to noise achievable, especially if the crystals have a rather low mosaic spread, since background noise will always accrue from the full energy range of the beam.

### 5.6. Backstops   

Backstops are some of the least exciting and most neglected components of the whole experimental setup. In practice, as beams have become smaller and more parallel the opportunity to record lower resolution data routinely has rarely been fully exploited. As noted above, a direct beam passing through air leads to noise from air scattering, so with in-air endstations the path from the crystal to the backstop should be absolutely minimal; however, beams are now so small that a perfectly designed beamstop should subtend a tiny angle. In reality, it is much easier to deal with this problem when the crystal environment is in a vacuum and the distance to the backstop can be relaxed. Meanwhile, there is perhaps still scope for innovation at traditional beamlines.

### 5.7. Detectors   

Detectors are now available with more, smaller pixels. This allows the spot to be isolated better from the background whilst still keeping the experimental setup relatively compact and affordable. As noted above, increased frame rates also allow more ideal data collection. The detector quantum efficiency of PILATUS detectors is now close to ideal (>90%) at the usual wavelengths (Donath *et al.*, 2013 ▸). Specialist integrating detectors are required for XFEL experiments and are still under active development (for example CSPAD and AGIPD; Wunderer *et al.*, 2016 ▸).

### 5.8. Data analysis   

Software packages generally perform rather well with ideal experiments; however, ideal software would also allow useful information to be extracted from data that otherwise would be thrown away. Progress in this direction has been partly stimulated by XFEL experiments, where data are fragmentary and may often come from the simultaneous excitation of multiple lattices (Ginn *et al.*, 2016 ▸); however, there has also been significant algorithm development for synchrotron data, for instance *BLEND* (Foadi *et al.*, 2013 ▸), which aims to help optimize the merging of multi-crystal data sets and is part of the overarching *DIALS* project. However, challenging projects still frequently require careful manual intervention.

### 5.9. Approaching the ideal   

In summary, the last ten years have shown how we might approach the ideal MX experiment, and modest progress has been made at most beamlines, with some extreme beamlines closing most of the gap to the ideal. The challenge is to roll out as much of this advance as possible to beamlines that will be robust, automated and very high throughput, so that they can impact most experiments, and seamlessly increase the scope of MX.

## 6. Threats to MX and a place in the future landscape for integrated structural biology   

### 6.1. Electron diffraction (ED)   

ED is a close cousin of X-ray diffraction; however, electrons interact far more strongly with biological specimens, so that even crystals that are the bread and butter of microfocus synchrotron beamlines are too thick for electron diffraction. At the energies currently used for electron diffraction (typically not more than 300 keV) a crystal of half a micrometre would already incur significant multiple scattering events (an electron diffracted more than once on its route through the crystal), making accurate measurement of the Bragg intensities problematic. ED is therefore the natural domain of nanocrystals. A major advantage of ED compared with MX is that roughly an order of magnitude more diffraction signal can be extracted before crippling radiation damage occurs. However, a drawback of ED is that although the scattering power increases for heavier atoms, it does so more slowly for electrons than X-rays, and anomalous scattering effects cannot be exploited. Thus, general vehicles for solving the phase problem in ED remain elusive. At present ED remains a niche activity, with Tamir Gonen, Tim Gruene and Jan-Peter Abrahams being the principal exponents (Rodriguez *et al.*, 2015 ▸, 2017 ▸), and most structures determined to date have been of peptides or rather small proteins (Clabbers *et al.*, 2017 ▸). In addition, characterization of electron scattering factors is still problematic (since electrons are charged, scattering is dramatically affected by the charge state of the atom; Wang & Moore, 2017 ▸). Overall this is an exciting area but is in its relative infancy, and there is an opportunity to transfer experience from MX to ED in both hardware (*e.g.* gonio­meters) and software, with the *DIALS* project already beginning to address the needs of ED. Ultimately, it would seem sensible to combine light and electron imaging, electron diffraction and X-ray diffraction in a single instrument capable of collecting the best possible diffraction data from crystals across the nanometre to micrometre size range.

### 6.2. Electron imaging (EM)   

EM can be accomplished easily through the application of magnetic lenses, and years of development have produced bright coherent electron sources, stable cryo-stages, phase plates, much-improved software and a new generation of direct electron detectors, which has resulted in the so-called ‘resolution revolution’ in single-particle analysis (SPA). For SPA many individual instances of macromolecules or complexes are imaged in assorted views, aligned and used to calculate a three-dimensional reconstruction of the particle (Kühlbrandt, 2014*b* ▸). The recent advances have impacted both the resolution, which can now, in favourable circumstances, be better than 2 Å (Bartesaghi *et al.*, 2015 ▸; Merk *et al.*, 2016 ▸), and the minimum particle size that can be analysed, for instance a 3.2 Å resolution structure of the 64 kDa haemoglobin tetramer was recently determined, aided by the increased contrast provided by the Volta phase plate (Khoshouei *et al.*, 2017 ▸). Although reasonably unbiased ways of estimating resolution are now available (Scheres & Chen, 2012 ▸), fully describing the electron scattering is still tricky (Wang & Moore, 2017 ▸), and there is a significant scope, and need, for better refinement protocols and validation metrics (Wlodawer *et al.*, 2017 ▸; Jakobi *et al.*, 2017 ▸). These trends will doubtless continue as detectors and microscope hardware continue to improve. For instance, the detective quantum efficiency of detectors, their frame rate and pixel count will improve and we can expect better automation. There may be value in exploring the use of higher energy machines (the current limit is 300 keV) and improved electron sources (for instance the use of cold-field emission-gun technology could increase source brightness approximately tenfold and potentially increase resolution through improved coherence). These advances will not only improve the scope of the method but will also allow increased ease of use, reliability and throughput. In particular, we expect the same trajectory of automated workflows that have revolutionized MX to increasingly impact electron microscopy, and the proper handling and preservation of metadata, pioneered in ISPyB, which is already being integrated with EM software analysis (la Rosa-Trevín *et al.*, 2016 ▸). In the medium term we can expect a rash of EM centres to spring up at synchrotrons, following the eBIC model established at Diamond, which currently houses four high-end machines (Stuart *et al.*, 2016 ▸; Clare *et al.*, 2017 ▸; Saibil *et al.*, 2015 ▸), and we might expect the number of high-end machines at such centres to be comparable to the number of MX beamlines that they sit alongside, to provide a comparable service to the community (Stuart *et al.*, 2016 ▸).

No doubt EM will be the method of choice for many interesting biological problems. What is less clear is how these machines will impact industrial research: there is still a long way to go before the throughput could even come within an order of magnitude of that achieved by projects such as XChem. Nevertheless, there will be increasing activity (although if unrealistic expectations are raised there may be a blip!). Finally, we should bear in mind that there is another approach to electron imaging: electron tomography (ET), in which a single instance of the object is rotated in the microscope to record a range of different views and a three-dimensional reconstruction is derived from this set of slices of the particle transform (Ortiz *et al.*, 2006 ▸; Briggs, 2013 ▸). This method, combined with ways of slicing eukaryotic cells that would otherwise be far too thick to image using current electron microscopes, will yield lower resolution information than SPA. Nonetheless, tomography should achieve molecular resolution (and where multiple instances of objects or parts of objects can be averaged should approach the point where atomic models can be constructed). This information will integrate beautifully with crystallographic analysis and SPA, and will open up opportunities for many basic science and industrial applications, including ‘the molecular pathology of the cell’, a term coined by Wah Chiu of Stanford, USA.

### 6.3. X-ray imaging   

X-ray imaging has the potential to zoom out to give a whole-cell view of eukaryotic cells and perhaps fragments of tissues and organoids. There are numerous imaging modes available. Full-field cryo-microscopy with X-rays at an energy where water is almost transparent is now offered at Diamond on beamline B24, using tomography to obtain three-dimensional reconstructions, in conjunction with correlative light microscopy (both wide field and super resolution; http://www.diamond.ac.uk/Beamlines/Imaging/B24.html). This uses samples attached to electron-microscope grids (often using cells that are grown on the grids, which are gold rather than copper to avoid cell toxicity). The resolution achieved is currently roughly an order of magnitude worse than can be obtained by cryo-electron tomography; however, the method has real potential value as a link in an unbroken chain from light microscopy through to the molecular and atomic detail of ET, SPA and MX. Indirect imaging by X-rays is also possible using the method of ptychography, where diffraction is recorded by scanning an X-ray beam over the sample and collecting data with the beam in a series of overlapping positions. The diffraction patterns then describe the transform of the illuminated volumes and are correlated since they contain common structure. Software can then reconstruct the scattering object. If this process is repeated as the sample is rotated, then a three-dimensional reconstruction can be obtained. This method is challenging, but using cryo-specimens it should ultimately be possible to match or exceed the resolution of full-field cryo X-ray microscopy. Finally XFELs, if they can deposit enough photons sufficiently rapidly into individual structures, might compete with EM single-particle analysis, although present sources are probably insufficiently bright.

## 7. Conclusions   

Crystallography is a routine tool for basic science and drug discovery, made generally accessible by synchrotron radiation. Where diffraction-quality crystals are available it can give greater detail more quickly than any other method. Its speed and power will continue to increase and the tedious phase problem is largely solved. We can expect software and hardware developments to make significant changes to the method and the direct involvement of the user in the experiment. Many changes will be incremental, but as data volumes increase there should be unexpected opportunities for the analysis of large data sets. Perhaps *PanDDA* (Pearce *et al.*, 2017 ▸) gives a flavour of this, and we should anticipate that artificial intelligence will provide significant unexpected impact. There is also scope for re-examining how we model disorder, thermal motion and solvent in crystals, and perhaps even a niche for divining information in the gaps between the Bragg peaks as beamlines push the background noise level further down and crystals are mounted closer to naked (Chapman *et al.*, 2011 ▸; Ayyer *et al.*, 2016 ▸). There is also the interesting question of how routine the experimental analysis of dynamic events in the crystal can be made, or whether in the long run other methods, especially those based on single molecules, will open up the fourth dimension of biology. In summary, we are likely to see crystallography no longer leading the charge on many headline-making complex structures, as electron-imaging methods take increasing ground in close to atomic resolution structure determination. As always, structural biologists will need to choose the right method for the job, and, perhaps even more than in the past, be prepared to combine methods. The future is bright. The future is integrative structural biology providing cellular snapshots which, in places, will approach atomic detail and, combined with results from reductionist molecular approaches, will provide a genuinely atomic view of cell biology.
